# Supplementary material for: Physiologically Based Pharmacokinetic Model of CYP2D6 Associated Interaction Between Venlafaxine and Strong Inhibitor Bupropion—The Influence of Age-Relevant Changes and Inhibitory Dose to Classify Therapeutical Success and Harm
Source: Pharmaceutics. 2025 Jan 31;17(2):179. doi: 10.3390/pharmaceutics17020179 (PMC11858960; doi:10.3390/pharmaceutics17020179)
Supplement: Supplementary file 1 [file pharmaceutics-17-00179-s001.zip › pharmaceutics-3338939-supplementary.pdf]

PHARMACEUTICS

Physiologically Based Pharmacokinetic Model of CYP2D6 Associated Interaction Between Venlafaxine and Strong Inhibitor Bupropion—Influence of Age-Relevant Changes and Inhibitory Dose to Classify Therapeutical Success and Harm

Ulrich R. Luecht [1], Wolfgang Scholz [2], Ann-Kathrin Geiben [3], Ekkehard Haen [3], Georg Hempel [1,*]

1 Department of Clinical Pharmacy, Institute of Pharmaceutical and Medical Chemistry, University of Muenster, Corrensstrasse 48, 48149 Muenster, Germany

2 ePrax GmbH, Dessauerstrasse 9, 80992 Munich, Germany

3 Clinical Pharmacology, The Departments of Pharmacology and Toxicology and for Psychatry and Psychotherapy, University of Regensburg, Universitätsstrasse 84, 93053 Regensburg, Germany

* Correspondence: georg.hempel@uni-muenster.de

1. Venlafaxine

For model evalution study data was used (Table S1). An output individual based on the published mean and a population of n=1000 individuals, based on the published ranges, were created. If there was a lack of demographic data, subjects were characterized based on a standard European (male, 30 years, 73 kg, 176 cm and a BMI of 23,57 kg/m²) in the age range of 20 to 50 years.

Table S1. Population characteristics extracted from the literature.

Fed status contains a previous standard high-fat breakfast immediately before taking the medicine. Indication of age, weight, height and BMI in mean value ± standard deviation (minimum value - maximum value)

study	application (duration)	dose [mg]	N	%female (♂/♀)	age [years]	height [cm]	weight [kg]	bmi [kg/m²]	status
Patat *et al.* [1]	IV (30 min) SD	10	16	0 (16/0)	23±2 (19-27)	177±7 (167-188)	71±5 (58-80)	-	-
Nichols *et al.* [2]	IV (1h) SD ODV	50	14	0 (14/0)	29.6±6.8 (18-45)	182.3±5.9	81.7±9.7	24.6±2.8	fast
Patat *et al.* [1]	PO SD	75	16	0 (16/0)	23±2 (19-27)	177±7 (167-188)	71±5 (58-80)	-	-
Troy *et al.* [3]	PO SD	75	12	7 (43/3)	30 (18-45)	-	77 (55-93)	-	Fast/fed
Troy *et al.* [3]	PO SD	150	15	7 (43/3)	30 (18-45)	-	77 (55-93)	-	Fast/fed
Troy *et al.* [4]	PO SD	150	24	50 (12/12)	27±6 (18-45)	-	74±11	-	-
Wright *et al.* [5]	PO SD	37.5	36	42 (21/15)	28±8.7 (18-49)	-	73±11.8 (49-99)	24.8±3 (19-30.2)	Fast/fed
Wright *et al.* [5]	PO SD	75	30	27 (22/8)	33.5±9.6 (18-48)	-	78.3±10.8 (59.4-98.0)	25.6±3.0 (20.8-29.9)	Fast/fed
Wright *et al.* [5]	PO SD	225	28	43 (16/12)	33.1±12.9 (18-55)	-	72.8±13.4 (51.7-98.0)	24.9±3.1 (20.6-31.2)	fed
Preskorn *et al.* [6]	PO SD	75	7	36	24 (18-55)	-	≥ 60	18-30	EM, fast
Preskorn *et al.* [6]	PO SD	75	7	36	24 (18-55)	-	≥ 60	18-30	PM, fast
Nichols *et al.* [7]	PO SD	75	7	14 (6/1)	30.1±10.4 (20-51)	180.6±7.9	82.2±11.7	18-30	EM, fast
Nichols *et al.* [7]	PO SD	75	7	43 (4/3)	28.3±8.9 (20-51)	177.3±13.7	78.2±14.1	18-30	PM, fast
Troy *et al.* [3]	PO MD (4d)	75	18	7 (43/3)	30 (18-45)	-	77 (55-93)	-	fast
Troy *et al.* [4]	PO MD (4d)	150	24	50 (12/12)	27±6 (18-45)	-	74±11	-	-
Wright *et al.* [5]	PO MD (7d)	225	33	12 (29/4)	26±8.1 (18-43)	-	80.7±13.7 (62.1-109)	25.9±3.7 (20.2-31.9)	fed

bmi body mass index; EM extensive metabolizer; IV intravenous; MD multiple dose; N number of subjects; PM poor metabolizer; PO peroral; SD single dose.

1.1. Literature-based modelling single and multiple peroral and single intravenous dose

Table S2. Evaluation of literature-based VEN/ODV single intravenous and peroral dose model regarding c_{max}, t_{max} and AUC.

study	character		c_{max} [ng/ml]			t_{max} [h]			AUC [ng*h/ml]				
			mod.	*lit.*	PE [%]	*mod.*	*lit.*	PE [%]	*mod.*	*lit.*	PE [%]	MPE [%]	MAPE [%]
Patat *et al.* [1]	10 mg IV	VEN	59.3	66.0	-10.0	0.5	0.5	0	179	182	-1.45	15.7	26.8
		ODV	14.4	18.0	-20.0	4.0	4.4	-9.09	324	401	-19.3	3.87	12.3
Nichols *et al.* [2]	50 mg IV	ODV	294	231.6	26.9	1.0	1.0	0	2641	2442	8.17	14.8	14.8
Wright *et al.* [5]	37.5 mg PO	VEN	26.9	24.76	8.64	-	-	-	513	365.59	40.3	21.8	23.0
									513	431.73*	18.8		
Wright *et al.* [5]	37.5 mg PO†	VEN	26.1	24.42	10.2	-	-	-	513	370.04	38.6	13.2	15.8
									513	449.12*	14.2		
Patat *et al.* [1]	75 mg PO	VEN	52.4	36.0	45.6	6.75	5.8	16.4	1046	564	85.5	84.0	84.0
		ODV	72.9	79.0	-7.70	13.8	8.3	65.7	2461	2196	12.1	-1.75	38.4
Troy *et al.* [3]	75 mg PO	VEN	51.7	45.0	14.8	6.75	6.5	3.85	1031	841	22.6	32.3	32.3
		ODV	70.0	65.0	7.50	13.8	10.7	28.5	2351	1967	19.5	12.2	19.6
Troy *et al.* [3]	75 mg PO†	VEN	50.9	45.0	13.1	8.25	6.7	23.1	1031	797	29.3	35.5	35.5
		ODV	69.8	69.0	1.13	15.0	11.0	36.4	2351	1982	18.6	10.1	17.1
Wright *et al.* [5]	75 mg PO	VEN	52.6	43.54	20.8	-	-	-	1015	649.48	56.3	25.6	25.6
									1018	705.20*	44.4		
Wright *et al.* [5]	75 mg PO†	VEN	51.5	42.47	21.3	-	-	-	1015	637.75	59.2	16.7	28.4
									1018	702.90*	44.8		
Troy *et al.* [3]	150 mg PO	VEN	104	89	16.6	6.75	6.1	10.7	2072	1834	13.0	42.9	44.9
		ODV	139	151	-7.8	13.8	11.5	19.6	4691	4418	6.18	-3.46	17.5
Troy *et al.* [3]	150 mg PO†	VEN	102	90	13.6	8.25	6.5	26.9	2072	1877	10.4	29.5	33.3
		ODV	139	154	-9.7	15.0	12.0	25.0	4692	4331	8.33	-5.14	27.5
Troy *et al.* [4]	150 mg PO	VEN	112	101	10.5	6.5	5.7	14.0	2078	1621	28.2	43.1	44.7
		ODV	160	167	-4.2	13.0	12.3	5.69	4558	4268	6.80	13.6	18.5
Wright *et al.* [5]	225 mg PO†	VEN	161	132.34	21.7	-	-	-	3222	2343.99	37.5	-6.86	23.4
									3222	2431.98*	32.5		
	**Mean**	**VEN**			**15.6**			**14.2**			**33.8**	**29.5**	**34.8**
		**ODV**			**-1.73**			**21.5**			**7.55**	**5.53**	**20.7**

* $AUC_{0-\infty}$ otherwise AUC_{0-t}; † with intake of food; AUC area under the curve; c_{max} maximal concentration; IV intravenous administration; lit. literature (observed); mod. model (predicted); MAPE mean absolute prediction error (precision); MPE mean prediction error (bias); ODV O-desmethylvenlafaxine; PE prediction error; PO peroral administration; t_{max} time of maximal concentration; VEN venlafaxine.

PBPK modeling of single intravenous dose VEN predicts well the plasma concentrations (Figure S1) found in the literature. MPE and MAPE of VEN and ODV show the good predictive ability of the intravenous model with high accuracy and precision (Table S2). Furthermore, the percentage of unchanged ODV excreted in the urine confirms that the integrated clearance of ODV via UDP-glucuronosyl transferase correctly reflects the metabolism of ODV. The modeling of single peroral doses using plasma concentration-time curves of VEN and ODV extracted from the literature reinforces the model's good predictive power (Figure S2).

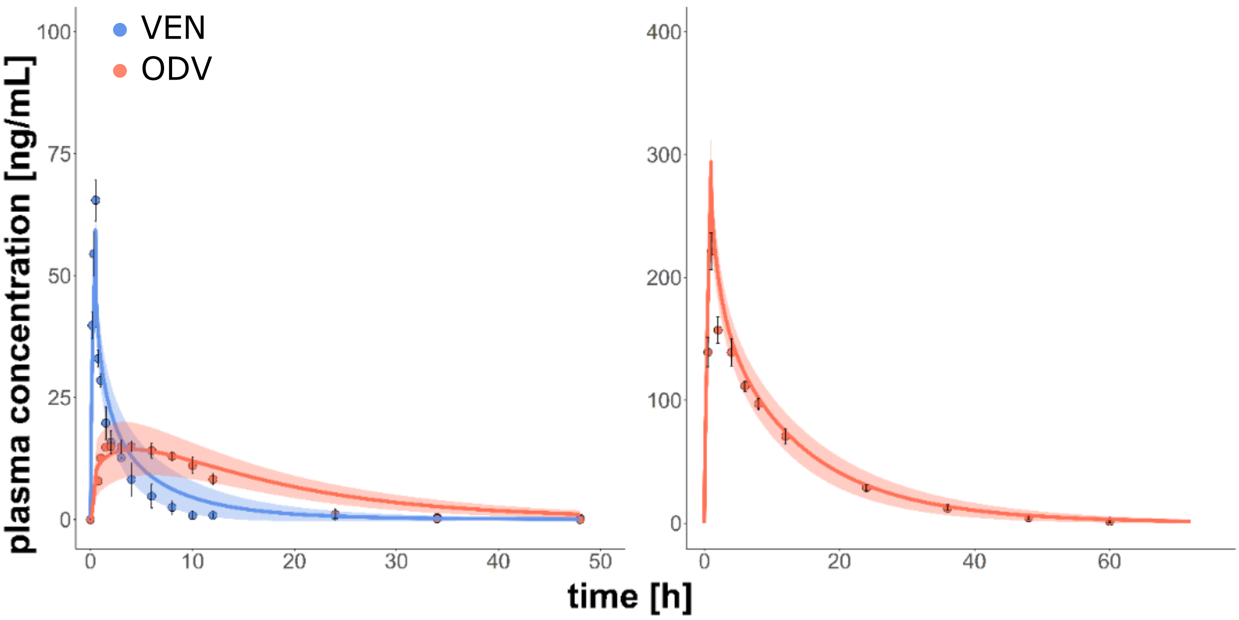

(a) **(b)**

Figure S1. Literature-based PBPK simulation of single intravenous administration.

(a) 10 mg VEN [Patat *et al.* [1], 1998]
(b) 50 mg ODV [Nichols *et al.* [2], 2012]

Solid lines represent the arithmetic mean of the simulation, the coloured areas the range of mean ± standard deviation, dots the mean values of plasma concentrations extracted from the literature. *ODV* O-desmethylvenlafaxine; *VEN* venlafaxine

Except for the simulations of the studies [1] and [5], the PEs of c_{max}, t_{max} and AUC of VEN and ODV are below 30% (Table S2), which is a sign of good predictivity. The estimation of the t_{max} of VEN occurs in all simulations with PEs between 3.85% and 26.9%, so that a correct simulation of the XR dosage form occurs.

The calculated concentration-time graphs of the multiple dose administration show that the literature-based modeling has good predictive ability with high accuracy and precision. The PE of the simulated c_{max}, t_{max} and AUC at steady-state of VEN and ODV are in the range of ± 20% (Table S3), which emphasizes besides MPE and MAPE the adequate predictivity, accuracy and precision of the PBPK-model.

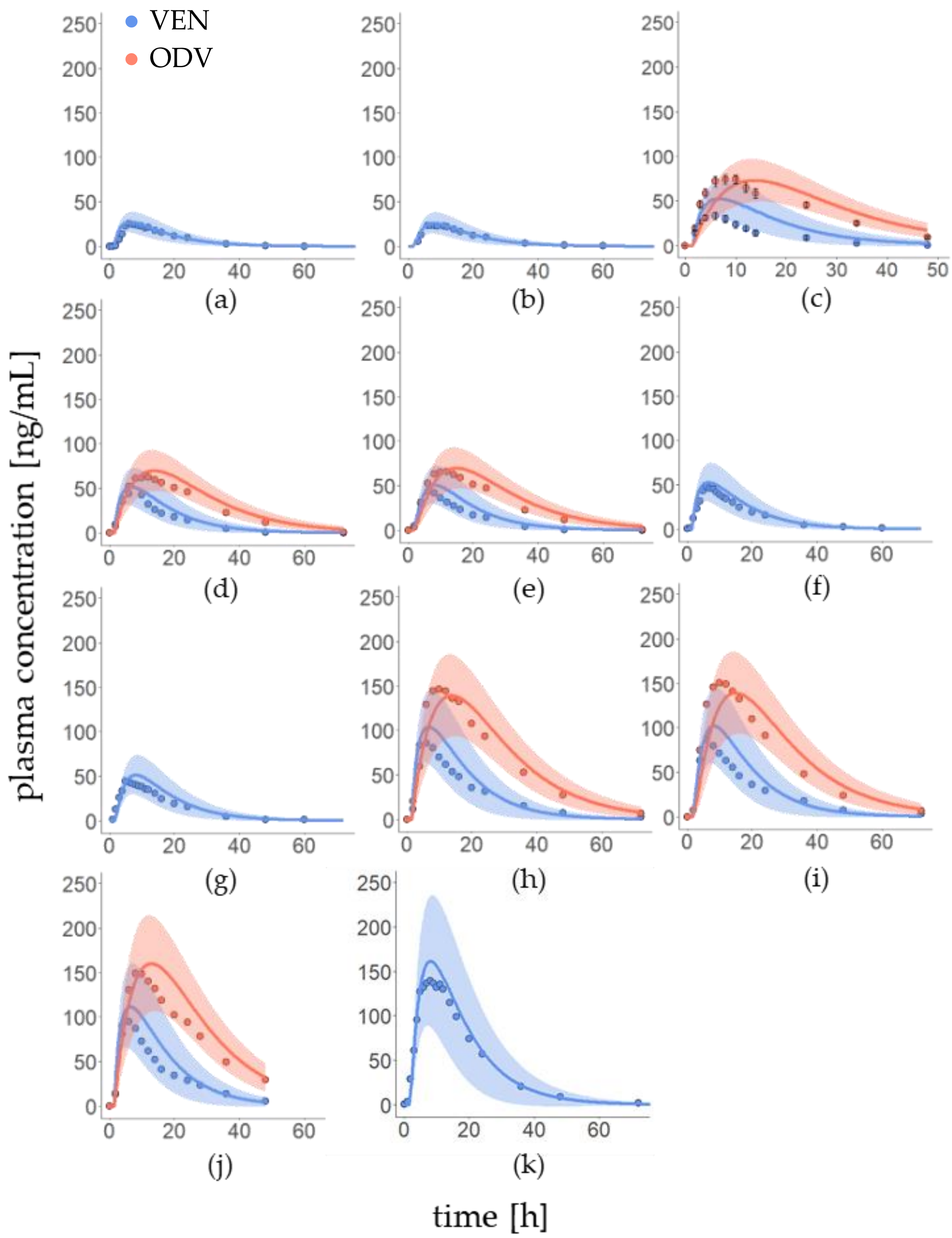

Figure S2. Literature-based PBPK simulation of single peroral administration of venlafaxine.

(a)	37.5 mg VEN	[Wright *et al.* [5], 2009]	(g)	75 mg VEN	[Wright *et al.* [5], 2009]
(b)	37.5 mg VEN	[Wright *et al.* [5], 2009]	(h)	150 mg VEN	[Troy *et al.* [4], 1997]
(c)	75 mg VEN	[Patat *et al.* [1], 1998]	(i)	150 mg VEN fasted	[Troy *et al.* [3], 1997]
(d)	75 mg VEN fasted	[Troy *et al.* [3], 1997]	(j)	150 mg VEN fed	[Troy *et al.* [3], 1997]
(e)	75 mg VEN fed	[Troy *et al.* [3], 1997]	(k)	225 mg VEN	[Wright *et al.* [5], 2009]
(f)	75 mg VEN	[Wright *et al.* [5], 2009]			

Solid lines represent the arithmetic mean, the coloured areas the range of mean ± standard deviation, points represent the mean values of plasma concentrations extracted from the literature.
ODV O-desmethylvenlafaxine; *VEN* venlafaxine

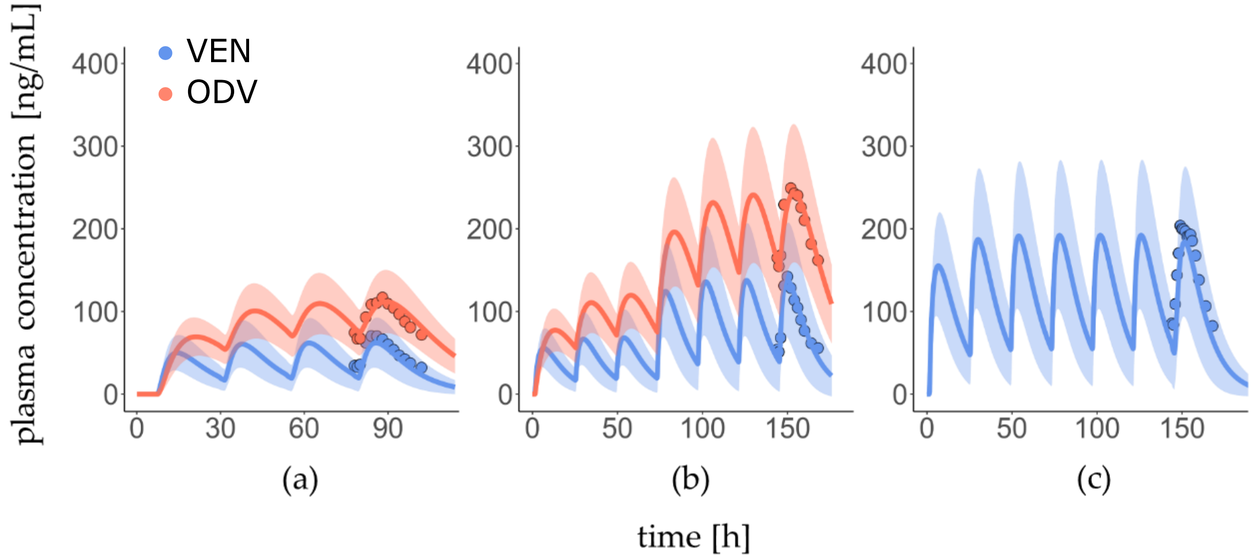

Figure S3. Literature-based PBPK simulation of peroral multiple administration of venlafaxine.

(a)	75 mg VEN once daily for four days	[Troy *et al.* [3], 1997].
(b)	150 mg VEN for four days after previous three-day administration of 75 mg	[Troy *et al.* [4], 1997].
(c)	225 mg VEN once daily for seven days	[Wright *et al.* [5], 2009]

Solid lines represent the arithmetic mean, the coloured areas the range of mean ± standard deviation, points represent the mean values of plasma concentrations extracted from the literature.
ODV O-desmethylvenlafaxine; *VEN* venlafaxine

Table S3. Evaluation of literature-based VEN/ODV multiple dose model regarding c_{max}, t_{max} and AUC.

Steady state simulations under fasting conditions.

study	character		c_{max} [ng/ml]			t_{max} [h]			AUC [ng*h/ml]			MPE [%]	MAPE [%]
			Mod.	*Lit.*	PE [%]	*Mod.*	*Lit.*	PE [%]	*Mod.*	*Lit.*	PE [%]		
Troy *et al.* [3]	75 mg XR once daily for 4 days	VEN	62.1	75.0	-17.0	7.75	7.2	7.64	1033	1220	-15.3	-19.3	19.3
		ODV	112	121	-7.6	11.3	9.9	13.6	2339	2251	3.89	5.43	9.09
Troy *et al.* [4]	150 mg XR once daily for 4 days	VEN	138	149	-7.5	6.0	5.4	11.1	2198	2222	-1.07	-3.18	8.34
		ODV	243	260	6.5	9.75	9.0	8.33	5011	5052	-0.82	-0.75	3.13
Wright *et al.* [5]	225 mg XR once daily for 7 days	VEN	184	198.02	-7.08	-	-	-	3817	3093.33	23.4	-17.9	17.9
	**Mean**	**VEN**			**-10.5**			**9.37**			**2.34**	**-13.5**	**15.2**
		**ODV**			**-0.55**			**11.0**			**1.54**	**2.34**	**6.11**

AUC area under the curve; c_{max} maximal concentration; *lit.* literature (observed); *mod.* model (predicted); *MAPE* mean absolute prediction error (precision); *MPE* mean prediction error (bias); *ODV* O-desmethyl-venlafaxine; *PE* prediction error; t_{max} time of maximal concentration; *VEN* venlafaxine; *XR* extended release.

1.2. Literature-based simulation of CYP2D6 phenotypes

After modification of k_{cat} values of CYP2D6 the PBPK models of the simulated CYP2D6 polymorphisms show good predictivity of the plasma concentrations of VEN and ODV (Figure S4). Overall, the calculated MPE and MAPE as well as calculated PE for cmax, tmax and AUC demonst-rate a precise and correct estimation of the model (Table S4).

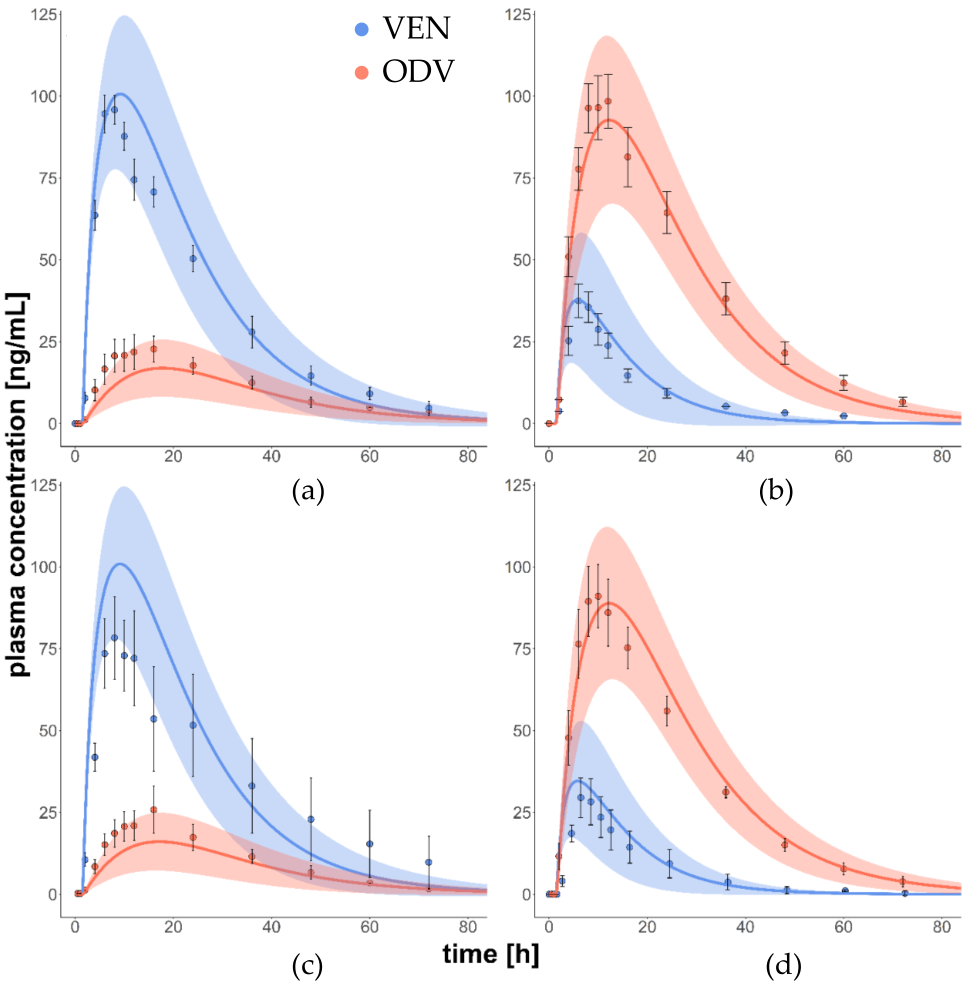

Figure S4. Literature-based PBPK simulation of peroral single dose in CYP2D6 EM and PM.

Simulation of 75 mg VEN in
(a) CYP2D6 PM [Preskorn *et al.* [6], 2009]
(b) CYP2D6 EM [Preskorn *et al.* [6], 2009],
(c) CYP2D6 PM [Nichols *et al.* [7], 2011]
(d) CYP2D6 EM [Nichols *et al.* [7], 2011].

Solid lines represent the arithmetic mean, the coloured areas the range of mean ± standard deviation, points represent the mean values of plasma concentrations extracted from the literature.
EM extensive metabolizers; ODV O-desmethylvenlafaxine; PM poor metabolizers; VEN venlafaxine

Table S4. Evaluation of literature-based VEN/ODV single peroral dose in CYP2D6 EM and PM model regarding c_{max}, t_{max} and AUC.

study	character		c_{max} [ng/ml]			t_{max} [h]			AUC [ng*h/ml]			MPE [%]	MAPE [%]
			Mod.	*Lit.*	PE [%]	*Mod.*	*Lit.*	PE [%]	*Mod.*	*Lit.*	PE [%]		
Preskorn *et al.* [6]	75 mg PM	VEN	101	98.6	2.1	9.25	7.0	32.1	2717	2548	6.65	8.05	34.5
		ODV	16.9	23.4	-28.0	17.8	16.0	10.9	676	844	-19.9	-30.2	30.2
Nichols *et al.* [7]	75 mg PM	VEN	101	79.8	26.5	9.0	8.0	12.5	2700	3054	-11.6	-0.71	54.7
		ODV	16.1	26.0	-38.0	17.3	12.0	43.8	627	791	-20.7	-28.3	28.3
	**Mean**	**VEN**			**14.3**			**22.3**			**-2.48**	**3.67**	**44.6**
		**ODV**			**-33.0**			**27.4**			**-20.3**	**-29.3**	**29.3**
Preskorn *et al.* [6]	75 mg EM	VEN	37.8	39.6	-4.6	5.75	6.0	-4.17	657	591	11.2	16.1	50.2
		ODV	92.7	104.3	-11.0	12.3	10.0	22.5	2879	3078	-6.46	-12.4	20.3
Nichols *et al.* [7]	75 mg EM	VEN	34.6	30.1	15.0	5.75	6.0	-4.17	598	518	15.5	67.7	74.6
		ODV	88.9	94.2	-5.6	12.3	10.0	22.5	2782	2589	7.44	1.31	8.57
	**Mean**	**VEN**			**5.20**			**-4.17**			**13.4**	**41.9**	**62.4**
		**ODV**			**-8.30**			**22.5**			**0.49**	**-5.55**	**14.4**

AUC area under the curve; c_{max} maximal concentration; *EM* CYP2D6 extensive metabolizer; *lit.* literature (observed); *mod.* model (predicted); *MAPE* mean absolute prediction error (precision); *MPE* mean prediction error (bias); *ODV* O-desmethylvenlafaxine; *PE* prediction error; *PM* CYP2D6 poor metabolizer; t_{max} time of maximal concentration; *VEN* venlafaxine.

1.3. Evaluation of pharmacokinetic parameters

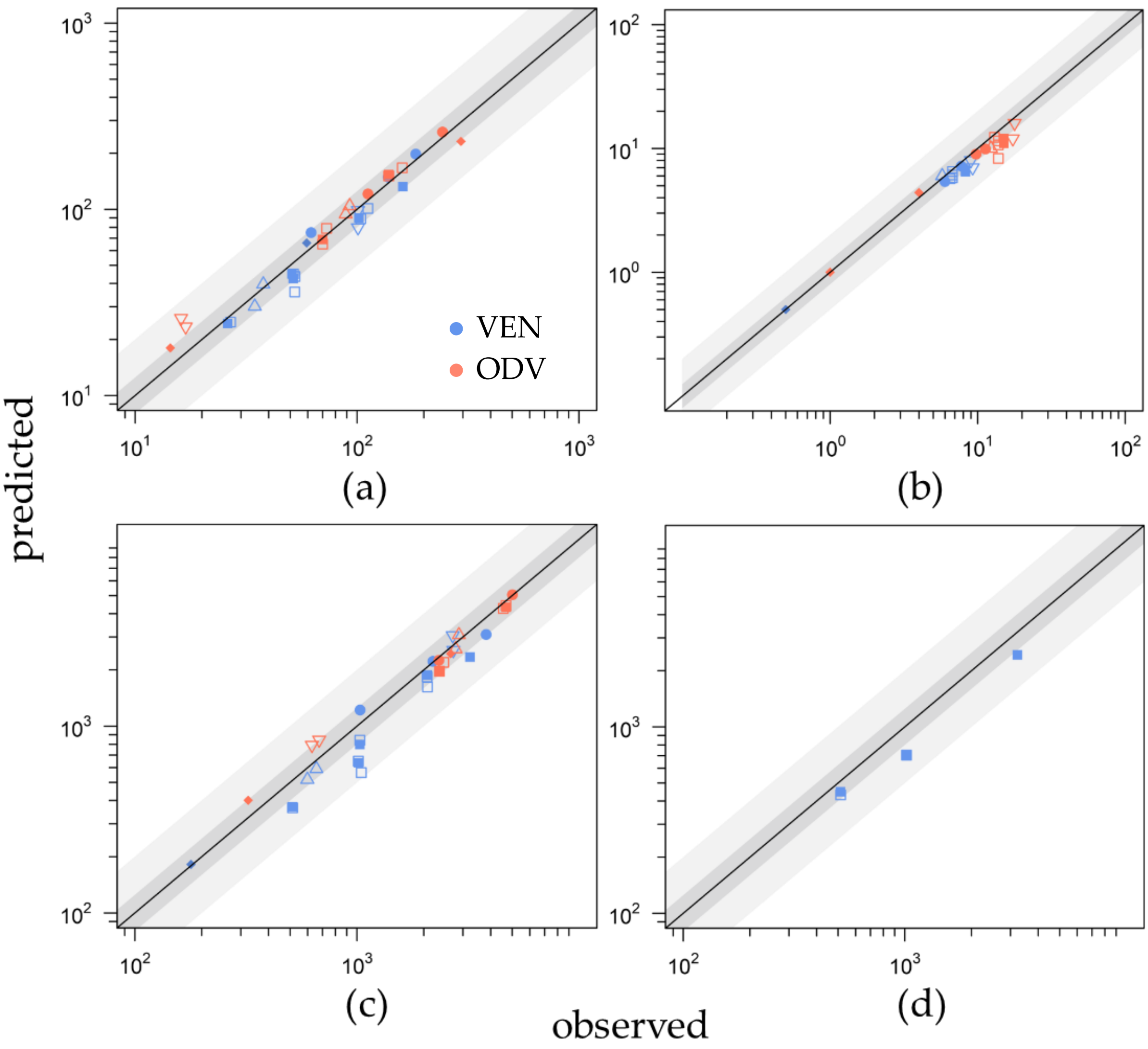

Figure S5. GOF-plot of pharmacokinetic parameters c_{max}, t_{max} and AUC of literature-based model VEN/ODV.

(a) c_{max} [ng/mL] (b) t_{max} [h] (c) AUC_{0-t} [ng*h/mL] (d) $AUC_{0-\infty}$ [ng*h/mL]

- ♦ single intravenous administration
- □ single peroral administration (fasting)
- ■ single peroral administration (fed)
- ▽ CYP2D6 poor metabolizer
- △ CYP2D6 extensive metabolizer
- ● multiple peroral administration (steady state)

2. Bupropion

Table S5. Summary of literature datasets used for PBPK modeling of BUP and its active metabolites.

All studies were conducted with healthy subjects. Age, height, weight and BMI are given as mean ± standard deviation (minimum value - maximum value). A meal is defined as a high-calorie breakfast consisting of eggs, bacon, buttered toast, fried potato dish and whole milk.

study	application (duration)	Dose regimen and administration	dose [mg]	N	$\%_{feminine}$ (♂/♀)	age [years]	height [cm]	weight [kg]	bmi [kg/m²]
patent by Oberegger *et al.* [8], 2006	p.o.	SD, with and without food intake	150 XR	32	31 (22 / 10)	-	-	-	-
	p.o.	SD, with and without food intake	300 XR	32	28 (23 / 9)	-	-	-	-
	p.o.	SD, without food intake	300 XR	35	46 (19 / 16)	-	-	-	-
	p.o. (13 days)	MD once daily, without food intake	300 XR	30	27 (22 / 8)	-	-	-	-
	p.o. (17 days)	MD once daily, without food intake	300 XR	49	25 (37 / 12)	-	-	-	-
Woodcock *et al.* [9], 2012	p.o.	SD, without food intake	300 XR	24	-	-	-	-	-
Paiement *et al.* [10], 2012	p.o.	SD, with and without food intake	450 XR	20	-	-	-	-	-
Benowitz *et al.* [11], 2013	p.o. (7 days)	MD once daily, without food intake	150 XR	42	38 (26 / 16)	33.1 (19 - 64)	-	76.1 (56.2 – 105.4)	26.3 (18.7 – 39.5)
Schmid *et al.* [12], 2015	p.o. (7 days)	MD, N.D.	300 XR	16	50 (8 / 8)	24.3 ± 2.2 (18 - 45)	-	-	22.7 ± 2.1 (20 - 25)
Connarn *et al.* [13], 2017	p.o.	SD, without food intake	150 XR 300 XR	33	51.5 (16 / 17)	25 - 55	167.5 (162 – 176.4)	79.8 (56.3 - 107)	25.9 (20.6 – 36.0)

bmi body mass index; *MD* multiple dose; *N* amount of participants; *N.D.* not defined; *p.o.* peroral; *SD* single dose; *XR* extended release

For model evaluation study data was used (Table S5). An output individual based on the published mean and a population of n=1000 individuals, based on the published ranges, were created. If there was a lack of demographic data, subjects were characterized based on a standard European (male, 30 years, 73 kg, 176 cm and a BMI of 23,57 kg/m²) in the age range of 20 to 50 years. The developed PBPK-model for single dose simulation of BUP (Figure S6) shows a good predictivity for the plasma concentrations of BUP and its metabolites. PE of t_{max} (BUP) yields values between -11.1% and +2.13% which shows that the simulated dosage form with extended drug release is accurately represented by the model (Table S6).

2.1. Single dose simulations

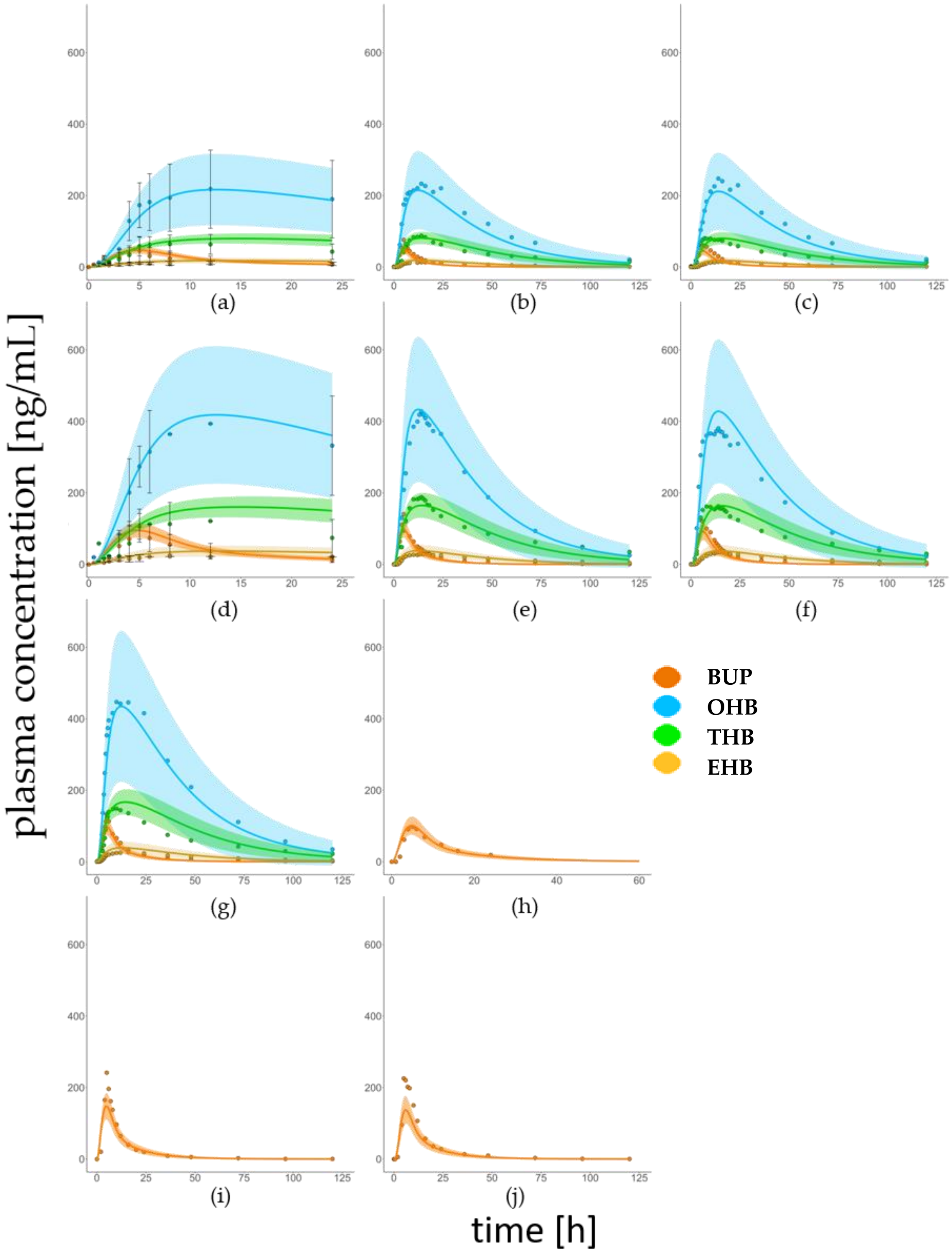

Figure S6. literature-based PBPK simulation of a single oral dose of bupropion.

150 mg BUP: (a) Connarn *et al.* [13], 2017; (b) fasting, Oberegger *et al. [8]*, 2006; (c) fed, Oberegger *et al.* [8], 2006; 300 mg BUP: (d) Connarn *et al.* [13], 2017; (e) fasting, Oberegger *et al*. [8], 2006; (f) fed, Oberegger *et al*. [8], 2006; (g) Oberegger *et al.* [8], 2006; (h) Woodcock *et al*. [9], 2012; 450 mg BUP: (i) fasting, Paiement *et al*. [10], 2012; (j) fed, Paiement *et al.* [10], 2012; Solid lines represent the arithmetic mean, the colored areas the range of mean ± standard deviation, dots the mean values of the plasma concentrations extracted from the literature.

Table S6. Comparison of pharmacokinetic parameters of BUP and its metabolites described and simulated in the literature after a single dose.

	study	dose [mg]	c_{max} [ng/mL]			t_{max} [h]			$AUC_{0-\infty}$ [ng*h/mL]			AUC_{0-t} [ng*h/mL]			MPE [%]	MAPE [%]
			Mod.	*Lit.*	PE [%]	*Mod.*	*Lit.*	PE [%]	*Mod.*	*Lit.*	PE [%]	*Mod.*	*Lit.*	PE [%]		
**BUP**	Connarn *et al.* [13]	150	47.5	61	-22.1	4.8	4.7	2.13	635	740	-14.2	-	-	-	19.8	28.2
	Oberegger *et al.* [8]	150	49.1	81.8	-40.0	4.8	5.13	-6.43	631	923	-31.7	630	865	-27.1	-10.7	34.5
	Oberegger *et al.* [8]†	150	45.6	75.8	-39.7	6	6.59	-8.95	631	966	-34.7	630	919	-31.4	-13.3	38.1
	Connarn *et al.* [13]	300	95.4	111	-14.1	4.8	4.8	0	1277	1356	-5.83	-	-	-	29.7	33.6
	Oberegger *et al.* [8]	300	99.3	151	-34.4	4.8	5.16	-6.98	1269	1833	-24.4	1268	1628	-22.2	-16.1	45.9
	Oberegger *et al.* [8]†	300	92.5	138	-33.1	6	6.16	-2.60	1269	1678	-30.8	1267	1775	-28.6	-15.0	51.5
	Oberegger *et al.* [8]	300	99.5	147	-32.3	4.8	5.16	-7.69	1321	1728	-23.6	1319	1677	-21.3	-5.55	49.6
	Woodcock *et al.* [9]	300	101	97.9	3.21	4.8	5.0	-4.0	-	-	-	1316	1036	27.1	32.9	41.2
	Paiement *et al.* [10]	450	148	254	-41.7	4.8	5.4	-11.1	1995	2274	-12.2	1992	2177	-8.49	1.90	39.6
	Paiement *et al.* [10]†	450	137	252	-45.4	6	6.25	-4.0	1995	2958	-32.6	1992	2851	-30.1	-0.05	62.1
	**mean**				**-30.0**			**-4.96**			**-23.3**			**-17.8**	**2.36**	**42.4**
**OHB**	Connarn *et al.* [13]	150	217	242	-10.5	12.6	12.2	3.28	13605	12100	12.4	-	-	-	-3.48	15.5
	Oberegger *et al.* [8]	150	215	246	9.18	12.3	15.2	-19.2	10529	13034	-19.2	10076	12612	-20.1	-5.73	20.6
	Oberegger *et al.* [8]†	150	212	252	-12.5	14	15.4	-8.97	10527	13050	-19.3	10060	12605	-20.2	0.95	23.5
	Connarn *et al.* [13]	300	418	402	4.09	12.6	10	26.0	26175	19700	32.9	-	-	-	24.3	43.7
	Oberegger *et al.* [8]	300	434	410	5.79	12.4	13.7	-9.56	21163	19853	6.60	20312	18939	7.25	18.8	20.5
	Oberegger *et al.* [8]†	300	428	449	-4.61	14.4	14.3	0.56	21159	20886	1.31	20282	19734	2.78	7.13	18.2
	Oberegger *et al.* [8]	300	435	479	-9.28	12.4	14.1	-11.8	21628	23499	-7.96	20700	22380	-7.51	20.0	37.2
	**mean**				**-2.55**			**-2.81**			**0.96**			**-7.56**	**8.85**	**25.6**
**THB**	Connarn *et al.* [13]	150	79.8	70.9	12.6	14.8	8.4	76.2	7496	3860	94.2	-	-	-	34.8	35.9
	Oberegger *et al.* [8]	150	81.2	88.4	-8.22	14.1	10.0	40.6	4697	5006	-6.19	4455	4686	-14.0	48.5	59.0
	Oberegger *et al.* [8]†	150	80.4	94.0	-14.4	15.8	11.9	32.3	4696	5360	-12.4	4446	4969	-10.5	0.01	23.0
	Connarn *et al.* [13]	300	161	144	11.6	14.9	9.2	62.0	15254	7350	108	-	-	-	43.7	43.7
	Oberegger *et al.* [8]	300	165	183	-9.57	14.2	9.94	42.9	9581	11696	-18.1	9089	9032	0.64	13.8	34.1
	Oberegger *et al.* [8]†	300	164	208	-21.3	15.9	12.3	29.7	9594	13281	-27.8	9083	9770	-7.02	13.5	32.6
	Oberegger *et al.* [8]	300	167	162	2.94	14.5	8.47	71.2	9851	9091	8.36	9332	7263	28.5	40.2	47.8
	**mean**				**-3.76**			**50.7**			**20.9**			**-0.48**	**27.8**	**39.4**
**EHB**	Connarn *et al.* [13]	150	18.2	13.8	31.9	13.1	12.4	5.65	1301	765	70.0	-	-	-	34.4	36.1
	Oberegger *et al.* [8]	150	19.0	14.2	34.0	12.7	15.6	-18.4	967	768	25.9	934	675	38.3	57.9	60.4
	Oberegger *et al.* [8]†	150	18.7	15.0	24.6	14.5	14.9	-2.55	967	816	18.5	932	723	29.0	39.7	43.6
	Connarn *et al.* [13]	300	37.3	24.8	50.5	13.2	10.3	28.2	2685	1860	44.4	-	-	-	38.3	43.2
	Oberegger *et al.* [8]	300	38.5	31.0	24.0	12.8	14.2	-9.60	1951	1868	4.45	1886	1635	15.4	35.2	37.2
	Oberegger *et al.* [8]†	300	38.1	35.8	6.45	14.4	14.7	-2.31	1951	2116	-7.81	1883	1803	4.43	18.5	31.5
	Oberegger *et al.* [8]	300	39.1	27.5	24.0	13.0	15.2	-14.7	2040	1614	26.4	1966	1442	36.3	61.9	67.9
	**mean**				**27.9**			**-1.96**			**26.0**			**24.7**	**40.8**	**45.7**

† after food intake; *$AUC_{0-\infty}$* area under the curve over the entire study period; *AUC_{0-t}* area under the curve within 24 hours; *BUP* bupropion; *c_{max}* maximum plasma concentration; *EHB* erythrohydrobupropion; *lit.* literature (observed); *MAPE* mean absolute prediction error (precision); *MPE* mean prediction error (bias); *mod.* model (predicted); *n_{urine}* amount of substance found in urine within 24 hours; *OHB* hydroxybupropion; *PE* prediction error; *THB* threohydrobupropion; *t_{max}* time of maximum plasma concentration

2.2. Multiple dose simulations

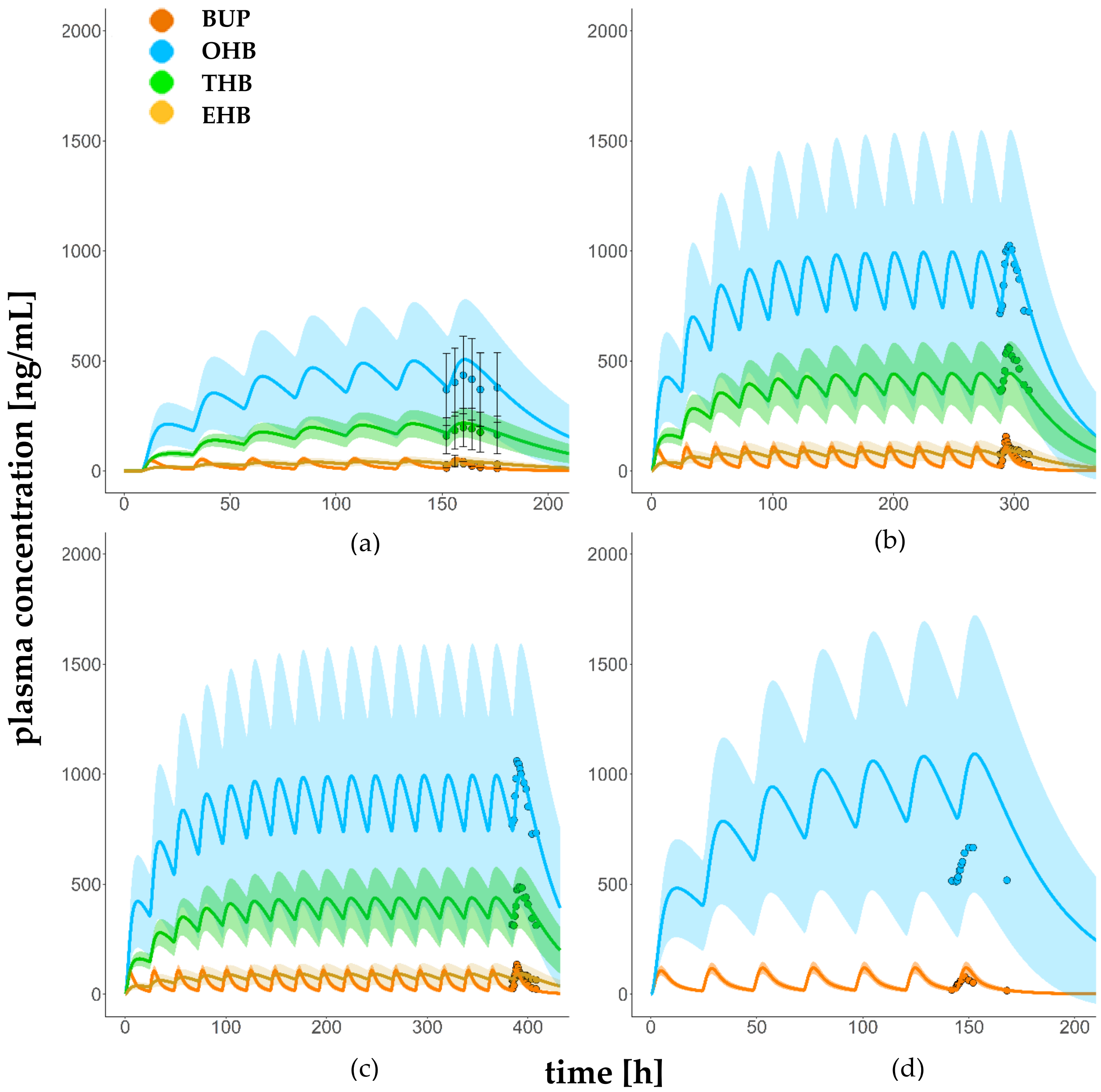

Figure S7. Literature-based PBPK simulation of multiple peroral administration of bupropion.

(a) 150 mg bupropion for 7 days [Benowitz *et al.* [11], 2013]; (b) 300 mg bupropion for 13 days [Oberegger *et al.* [8], 2006]; (c) 300 mg bupropion for 17 days [Oberegger *et al.* [8], 2006]; (d) 300 mg bupropion for 7 days [Schmid *et al.* [12], 2015]; Solid lines represent the arithmetic mean. the colored areas the range of mean ± standard deviation. dots the mean values of the plasma concentrations extracted from the literature.

Except for one value of AUC_{0-t} (OHB), all pharmacokinetic parameters of BUP, OHB, THB and EHB are within PE ±50% (Table S7). The illustration of plasma concentration-time curves also indicates a good predictive power of the created model (Figure S7). Excluding the results of the simulation of the study by Schmid *et al.* [12], MPE and MAPE are below ±30%, which is a prove of good predictivity (Table S7).

Table S7. Comparison of pharmacokinetic parameters of BUP and its metabolites described in the literature and simulated after multiple doses.

	study	dose [mg]	t [days]	c_{max} [ng/mL]			t_{max} [h]			AUC_{0-t} [ng*h/mL]			n_{Urin} [nmol]			MPE [%]	MAPE [%]
				mod.	*lit.*	PE [%]	*mod.*	*lit.*	PE [%]	*mod.*	*lit.*	PE [%]	*mod.*	*lit.*	PE [%]		
**BUP**	Benowitz *et al.* [11]	150	7	54.5	58	-6.07	-	-	-	647	685	-5.62	2203	2087	1.06	1.64	12.7
	Oberegger *et al.* [8]	300	13	110	168	-34.3	4.7	4.9	-4.08	1257	1612	-22.0	-	-	-	-17.8	26.1
	Oberegger *et al.* [8]	300	17	108	149	-27.3	4.7	4.92	-4.47	1234	1464	-15.7	-	-	-	-11.2	22.6
	Schmid *et al.* [12]	300	7	92.9	93.2	-0.28	4.75	4.75	-6.86	1337	1030	29.8	-	-	-	14.6	37.5
	**mean**					**-17.0**			**-5.14**			**-3.38**			**1.06**	**-3.19**	**24.7**
**OHB**	Benowitz *et al.* [11]	150	7	507	464	9.18	-	-	-	10883	9524	14.3	3706	3672	1.01	12.9	12.9
	Oberegger *et al.* [8]	300	13	997	1096	-8.98	8.7	7.3	19.2	21331	20825	2.43	-	-	-	0.93	4.16
	Oberegger *et al.* [8]	300	17	996	1111	-10.4	8.7	6.61	31.6	21315	21256	0.28	-	-	-	-2.07	5.16
	Schmid *et al.* [12]	300	7	1092	748	46.0	8.0	7.3	9.59	23269	14490	60.6	-	-	-	54.4	54.4
	**mean**					**8.95**			**20.1**			**19.4**			**1.01**	**16.5**	**19.2**
**THB**	Benowitz *et al.* [11]	150	7	218	208	4.68	-	-	-	4797	4209	14.0	38803	38376	1.11	10.4	10.4
	Oberegger *et al.* [8]	300	13	444	585	-24.1	8.1	7.83	3.45	9752	10988	-11.2	-	-	-	-10.6	10.8
	Oberegger *et al.* [8]	300	17	439	525	-16.4	8.1	7.74	4.65	9638	9639	0.01	-	-	-	1.19	8.90
	**mean**					**-11.9**			**4.05**			**0.94**			**1.11**	**0.33**	**10.0**
**EHB**	Benowitz *et al.* [11]	150	7	44.3	38	16.6	-	-	-	961	772	24.4	5484	4872	12.6	27.9	27.9
	Oberegger *et al.* [8]	300	13	92.8	109	-14.9	8.1	8.4	-3.23	2002	2146	-6.68	-	-	-	-6.95	6.95
	Oberegger *et al.* [8]	300	17	92.2	97.1	-5.07	8.1	8.3	-2.53	1992	1875	6.23	-	-	-	6.48	6.60
	**mean**					**-1.12**			**-2.88**			**7.98**			**12.6**	**9.14**	**13.8**

AUC_{0-t} area under the curve within 24 hours; *BUP* bupropion; *c_{max}* maximum plasma concentration; *EHB* erythrohydrobupropion; *lit.* literature (observed); *mod.* model (predicted); *MAPE* mean absolute prediction error (precision); *MPE* mean prediction error (bias); *n_{urine}* amount of substance found in urine within 24 hours; *OHB* hydroxybupropion; *PE* prediction error; *THB* threohydrobupropion; *t_{max}* time of maximum plasma concentration

2.3. Model Evaluation

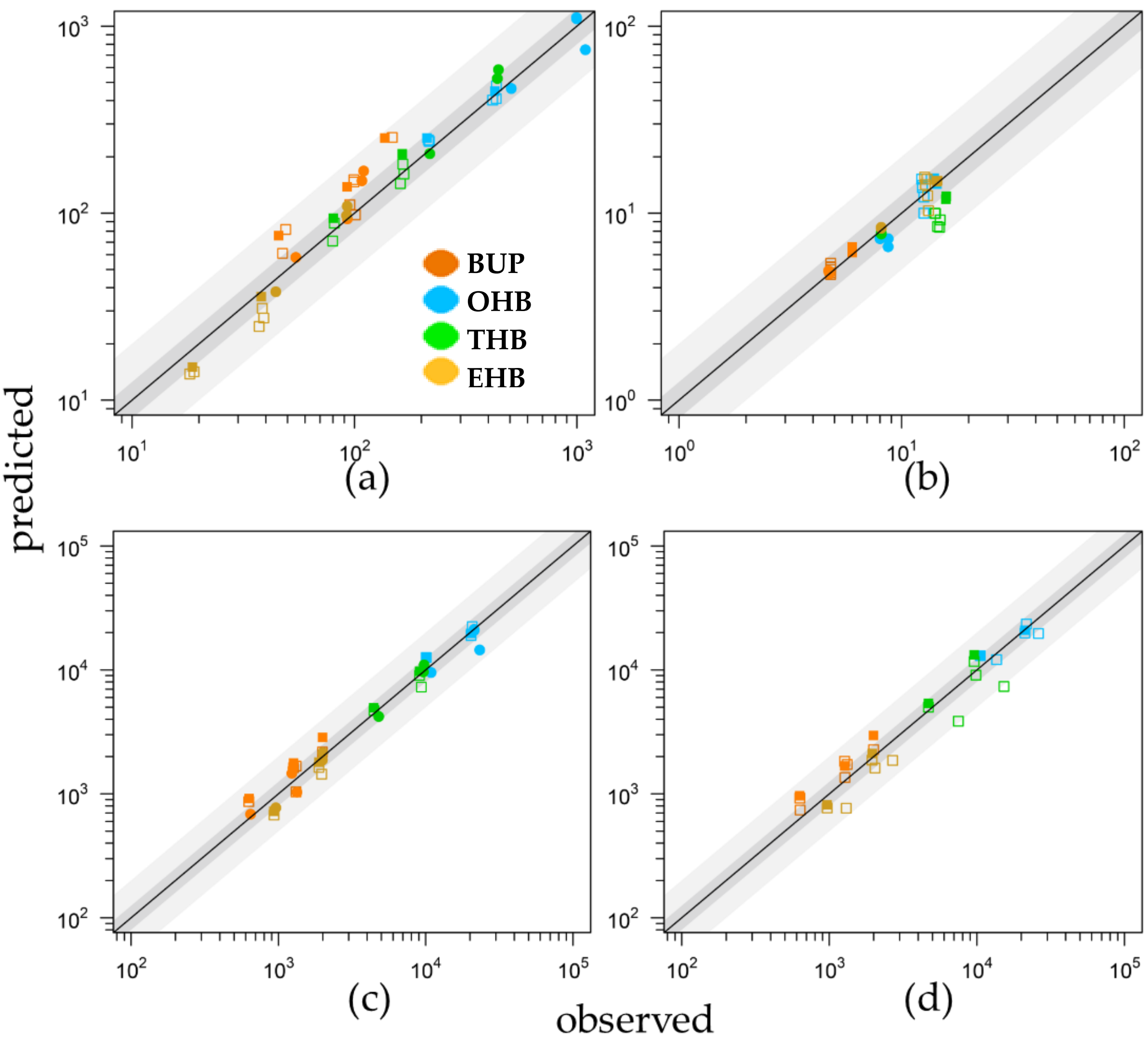

Figure S8. GOF-plot of c_{max}, t_{max} and AUC

(a) c_{max} [ng/mL] (b) t_{max} [h] (c) $AUC_{0\text{-}t}$ [ng*h/mL] (d) $AUC_{0\text{-}\infty}$ [ng*h/mL]

☐ single peroral administration (fasting)

■ single peroral administration (fed)

● multiple peroral administration (steady state)

The GOF-plots (Figure S8) illustrate that only one value of AUC (THB) is outside the two-fold error range, so there is a good predictive power of the built model. For the single dose simulations 67.7% of all cmax, 71.0& of all t_{max}, 60.9% of all $AUC_{0\text{-}t}$ and 60% of all $AUC_{0\text{-}\infty}$ values are within the 1.25 fold error range. For multiple dose simulations 71.1% of all c_{max}, 75.6% of t_{max} and 70.3% of $AUC_{0\text{-}t}$ are within the 1.25 fold error range respectively.

3. DDI – Age-relevant changes

To highlight the age-related differences more precisely, a statistical analysis was carried out. For this purpose, a new population was first created based on the KONBEST data, which can best represent the demographics of the patients who were treated with venlafaxine and bupropion. The following key data were used for the starting individual (ranges): female patient (57%), 51 years old (20-78 years old), 170 cm (152-193 cm), 85 kg (52-140 kg), 29.6 kg/m². The study simulated n=1000 individuals. Statistical analysis was performed using the Wilcoxon rank sum test. The statistical analysis shows that both without and with BUP comedications, AUC and cmax show statistically significant higher trends (red highlighted) in older patients than in younger patients (Figure S9, Figure S10).

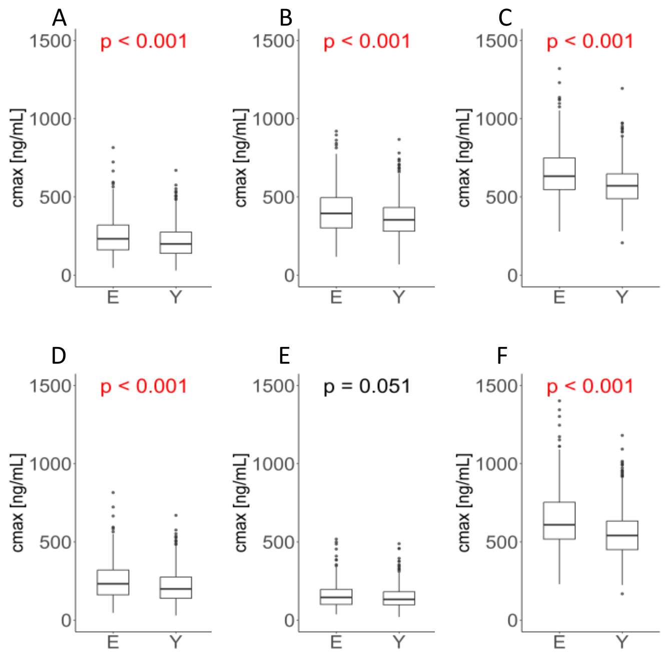

Figure S9. Boxplots of C_{max} in elderly (E) and young (Y) patients before BUP comedication (A-C) and during BUP comedication (D-F).

A, D C_{max} (VEN)
B, E C_{max} (ODV)
C, F C_{max} (AM)

AM active moiety, BUP bupropion, ODV O-desmethylvenlafaxine, VEN venlafaxine.

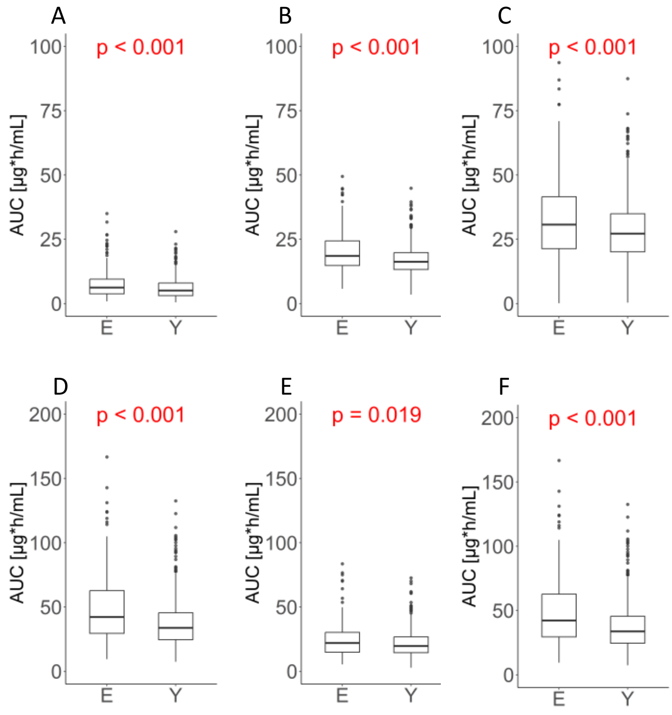

Figure S10. Boxplots of AUC in elderly (E) and young (Y) patients before BUP comedication (A-C) and during BUP comedication (D-F).

A, D AUC (VEN)
B, E AUC (ODV)
C, F AUC (AM)

AM active moiety, BUP bupropion, ODV O-desmethylvenlafaxine, VEN venlafaxine.

4. DDI venlafaxine and itraconazole

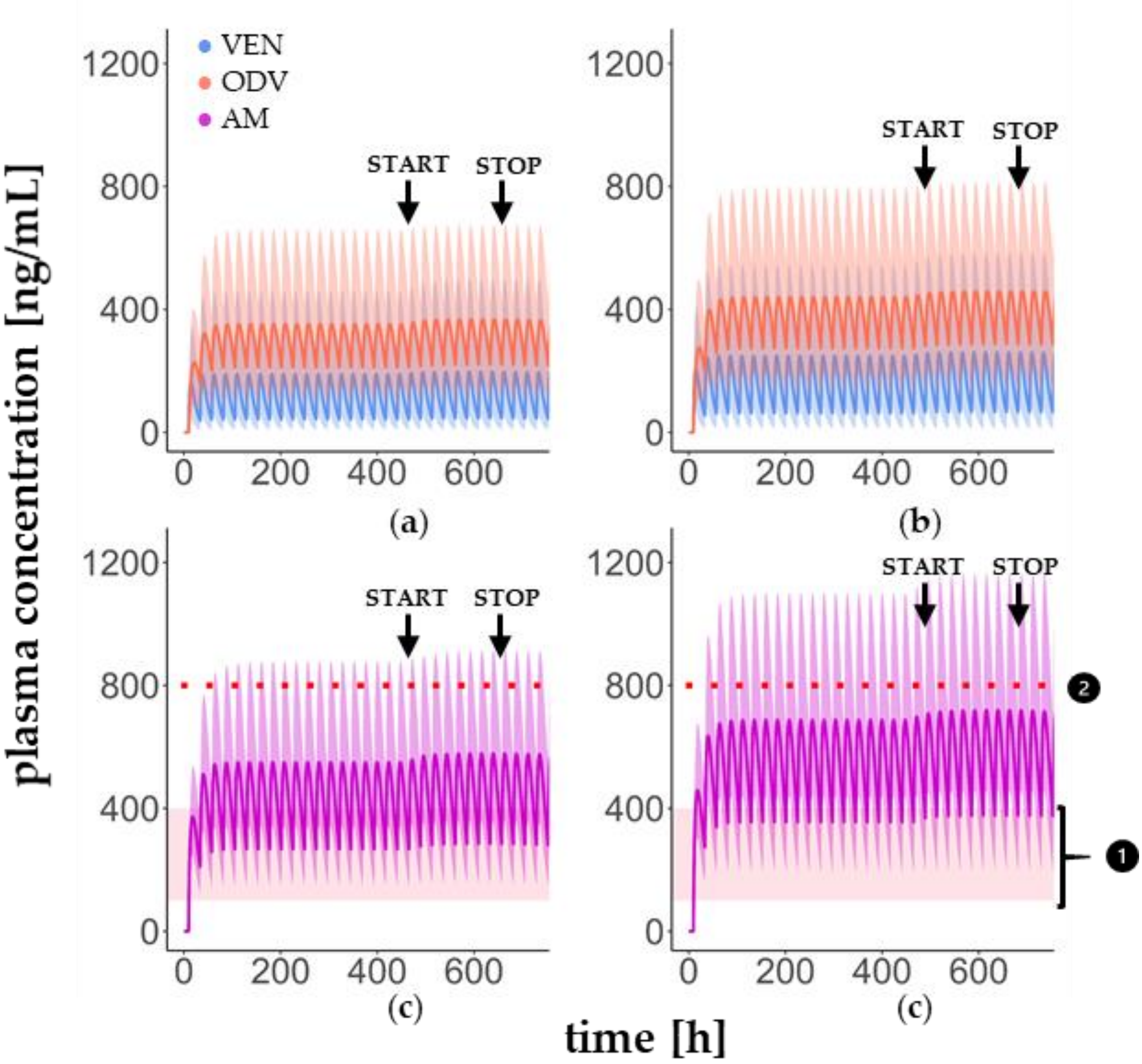

Figure S11. Plasma concentration time curves of VEN, ODV and AM before, during and after ITRA co-medication in young (a,c) and elderly (b,d) patients.

Administration protocol contains start and stop of 100 mg ITRA twice daily. Plasma concentration time curves of ITRA and Hydroxy-ITRA are not illustrated to avoid confusion. To interpretate the influence of DDI, parameters of TDM (❶ therapeutic reference range (AM 100-400 ng/ml), ❷ toxic range (AM > 800 ng/ml)) are used. *AM* active moeity; *ITRA* itraconazole; *ODV* O-desmethyl-venlafaxine; *TDM* therapeutic drug monitoring; *VEN* venlafaxine

If the AUC of the median of VEN, ODV and AM is compared with and without ITRA comedication in younger patients, an increase of AUC by 5.34%, 5.31% and 5.85% results. In older patients, the PBPK interaction model calculates an AUC increase of 5.26%, 5.23% and 5.64% after multiple doses of 225 mg venlafaxine with ITRA co-medication (Figure S11).

References

1. Patat A, Troy S, Burke J, et al. Absolute bioavailability and electroencephalographic effects of conventional and extended-release formulations of venlafaxine in healthy subjects. J Clin Pharmacol 1998;38(3):256–67.
2. Nichols AI, A Behrle J, Richards LS. The Absolute Bioavailability of Desvenlafaxine in Healthy Subjects. J Bioequiv Availab 2012;04(02).
3. Troy SM, Dilea C, Martin PT, Leister CA, Fruncillo RJ, Chiang ST. Pharmacokinetics of once-daily venlafaxine extended release in healthy volunteers. Current Therapeutic Research 1997;58(8):504–14.
4. Troy SM, Dilea C, Martin PT, Rosen AS, Fruncillo RJ, Chiang ST. Bioavailability of once-daily venlafaxine extended release compared with the immediate-release formulation in healthy adult volunteers. Current Therapeutic Research 1997;58(8):492–503.
5. Wright CW, Aikman MS, Werts E, Seabolt J, Haeusler J-MC. Bioequivalence of single and multiple doses of venlafaxine extended-release tablets and capsules in the fasted and fed states: four open-label, randomized crossover trials in healthy volunteers. Clinical Therapeutics 2009;31(11):2722–34.
6. Preskorn S, Patroneva A, Silman H, et al. Comparison of the pharmacokinetics of venlafaxine extended release and desvenlafaxine in extensive and poor cytochrome P450 2D6 metabolizers. Journal of Clinical Psychopharmacology 2009;29(1):39–43.
7. Nichols AI, Focht K, Jiang Q, Preskorn SH, Kane CP. Pharmacokinetics of venlafaxine extended release 75 mg and desvenlafaxine 50 mg in healthy CYP2D6 extensive and poor metabolizers: a randomized, open-label, two-period, parallel-group, crossover study. Clin. Drug Investig. 2011;31(3):155–67.
8. Oberegger W, Eradiri O, Zhou F, Maes P. Modified-release tablet of bupropion hydrochloride: Patent US007537784B2, 2006.
9. Woodcock J, Khan M, Yu LX. Withdrawal of generic budeprion for nonbioequivalence. N Engl J Med 2012;367(26):2463–5.
10. Paiement N, Noonan PK, Gonzalez MA, Zerbe H. Steady State Plasma Levels of Bupropion After Administration of 3× 150 Mg Extended Release Reference Tablets and Switching to 1x450 Mg Extended Release 450ER Tablets. International Journal of Clinical Pharmacology & Toxicology 2012;1(1):26–31.
11. Benowitz NL, Zhu AZX, Tyndale RF, Dempsey D, Jacob P. Influence of CYP2B6 genetic variants on plasma and urine concentrations of bupropion and metabolites at steady state. Pharmacogenetics and Genomics 2013;23(3):135–41.
12. Schmid Y, Rickli A, Schaffner A, et al. Interactions between bupropion and 3,4-methylenedioxymethamphetamine in healthy subjects. J Pharmacol Exp Ther 2015;353(1):102–11.
13. Connarn JN, Flowers S, Kelly M, et al. Pharmacokinetics and Pharmacogenomics of Bupropion in Three Different Formulations with Different Release Kinetics in Healthy Human Volunteers. AAPS J 2017;19(5):1513–22.
